# Supplementary material for: Hindsight is 2020 vision: a characterisation of the global response to the COVID-19 pandemic
Source: BMC Public Health. 2020 Dec 7;20:1868. doi: 10.1186/s12889-020-09972-z (PMC7719727; doi:10.1186/s12889-020-09972-z)
Supplement: Supplementary file 1 — Additional file 1 Supplementary material. [file 12889_2020_9972_MOESM1_ESM.pdf]

Supplementary Material for "Hindsight is 2020 vision: a characterisation of the global response to the COVID-19 pandemic"

David J. Warne[1,2], Anthony Ebert[3], Christopher Drovandi[1,2], Wenbiao Hu[4], Antonietta Mira[3,5], and Kerrie Mengersen[1,2]

[1]School of Mathematical Sciences, Science and Engineering Faculty, Queensland University of Technology, Brisbane, Australia
[2]Australian Research Council Centre of Excellence for Mathematical and Statistical Frontiers
[3]Institute of Computational Science, Università della Svizzera italiana, Lugano, Switzerland
[4]School of Public Health and Social Work, Institute of Health and Biomedical Innovation, Queensland University of Technology, Birsbane, Australia
[5]Dipartimento di Scienza e Alta Tecnologia, Università dell´Insubria, Varese, Italy

November 20, 2020

Contents

Appendix A Stochastic model details **2**
A.1 Model formulation 2
A.2 Approximate stochastic simulation 2

Appendix B Bayesian analysis **4**
B.1 Approximate Bayesian computation 4
B.2 Sequential Monte Carlo sampling 4
B.3 Point estimate computation 6

Appendix C Marginal posterior comparisons **7**

Appendix D Example posterior predictive distributions **17**

Appendix E Sensitivity analysis **28**

Appendix A Stochastic model details

In the main manuscript, we present our model as a nonlinear system of ordinary differential equations (ODEs) for ease of discourse. Due the variability in daily reported cases, however, it is important to take into account stochasticity in our analysis.

A.1 Model formulation

We implement our model as a stochastic compartmental epidemiological model consisting of the compartments, S (susceptible), I (undocumented infected), A (confirmed active), R (confirmed recovered), D (confirmed death), and R^u (undocumented recovered). Transition events between compartments are

$$\mathcal{E}_1 : S \to I, \quad \mathcal{E}_2 : I \to A, \quad \mathcal{E}_3 : A \to R, \quad \mathcal{E}_4 : A \to D, \quad \text{and} \quad \mathcal{E}_5 : I \to R^u.$$

Let $\mathbf{X}_t = [S_t, I_t, A_t, R_t, D_t, R_t^u]^{\mathrm{T}}$ be the state vector of sub-population counts at time $t > 0$, and assume a well-mixed population of size P. Conditional on the state $\mathbf{X}_t$, the waiting time to the next occurrence of event $\mathcal{E}_j$ is assumed to be exponentially distributed with rate parameter $h_j(\mathbf{X}_t)$, where $h_j(\mathbf{X}_t)$ is the hazard function for $\mathcal{E}_j$. The hazard functions can be interpreted as the instantaneous rate of events conditional on the current state.

The hazard functions of our model are:

$$h_1(\mathbf{X}_t) = \left(\alpha_0 + \frac{\alpha}{1 + U(A_t, R_t, D_t)^n}\right) \frac{S_t I_t}{P}, \quad h_2(\mathbf{X}_t) = \gamma I_t,$$
$$h_3(\mathbf{X}_t) = \beta A_t, \quad h_4(\mathbf{X}_t) = \delta A_t, \quad \text{and} \quad h_5(\mathbf{X}_t) = \eta\beta I_t,$$

where $U(A, R, D) = w_A A_t + w_R R_t + w_D D_t$ is the reporting function of observables with weights $w_A > 0$ $w_R > 0$ and $w_D > 0$. Model parameters related to the event rates are the transmission rates $\alpha_0 > 0$, $\alpha > 0$, the response slope $n \geq 0$, identification rate $\gamma > 0$, case recovery rate $\beta > 0$, case fatality rate $\delta > 0$, and the latent recovery rate scale factor $\eta > 0$.

Should event j occur, the state vector is updated by adding the state change vector ν_j. For our model we have, $\boldsymbol{\nu}_1 = [-1, 1, 0, 0, 0, 0]^{\mathrm{T}}$, $\boldsymbol{\nu}_2 = [0, -1, 1, 0, 0, 0]^{\mathrm{T}}$, $\boldsymbol{\nu}_3 = [0, 0, -1, 1, 0, 0]^{\mathrm{T}}$, $\boldsymbol{\nu}_4 = [0, 0, -1, 0, 1, 0]^{\mathrm{T}}$, and $\boldsymbol{\nu}_5 = [0, -1, 0, 0, 0, 1]^{\mathrm{T}}$. The resulting stochastic process, $\{\mathbf{X}_t\}_{t \geq 0}$, is a discrete-state, continuous-time Markov process that can be described by the Kurtz random time-change representation [6],

$$\mathbf{X}_t = \mathbf{X}_0 + \sum_{j=1}^{5} Y_j \left(\int_0^t h_j(\mathbf{X}_s) \mathrm{d}s\right) \boldsymbol{\nu}_j,$$

where $\mathbf{X}_0$ is the initial state vector, ν_j is the state change that occurs under event j, and $Y_j(\cdot)$ is a inhomogeneous Poisson process for event j.

A.2 Approximate stochastic simulation

While exact realisations of this process can be generated using event-based simulation [3, 4], this is prohibitive within an approximate Bayesian computational setting with large

population sizes and event numbers. Therefore, we apply a first order approximation to the integral over the interval $[t, t + \tau)$ to obtain the tau-leaping approximation [5],

$$\mathbf{X}_{t+\tau} = \mathbf{X}_t + \sum_{j=1}^{5} Y_j \boldsymbol{\nu}_j + \mathcal{O}(\tau),$$

where $Y_j \sim \text{Poisson}\,(h_j(\mathbf{X}_t)\tau)$ counts the number of times event j occurs in the interval $[t, t + \tau)$. Simulations proceed as per Algorithm 1. For our simulations we use $\tau = 1$ (days), and initial condition $\mathbf{X}_0 = [P - \kappa A_0 - (A_0 + R_0 + D_0), \kappa A_0, A_0, R_0, D_0, 0]^{\mathrm{T}}$, where A_0, R_0 and D_0 come from the Johns Hopkins University COVID-19 data, and κ is the relative number of unobserved cases at $t = 0$.

Algorithm 1 Tau-leaping approximate stochastic simulation method

Initialise $t \leftarrow 0$; $\mathbf{X}_0 \leftarrow [P - \kappa A_0 - (A_0 + R_0 + D_0), \kappa A_0, A_0, R_0, D_0, 0]^{\mathrm{T}}$;
while $t + \tau < T$ **do**
    for $j \in \{1, 2, \ldots, 5\}$ **do**
        $Y_j \sim \text{Poisson}(h_j(\mathbf{X}_t)\tau)$;
    end for
    for $j \in \{1, 2, \ldots, 5\}$ **do**
        $\mathbf{X}_{t+\tau} \leftarrow \mathbf{X}_t + Y_j \boldsymbol{\nu}_j$
    end for
    $t \leftarrow t + \tau$;
end while

Appendix B Bayesian analysis

We apply Bayesian inference to quantify uncertainty in the model parameters associated with event rates and community response, $\theta = [\alpha_0, \alpha, \beta, \gamma, \delta, \eta, n, \kappa, w_A, w_R, w_D]$, for country i using Johns Hopkins University data $\mathcal{D}_i = [\{C_{t,i}, R_{t,i}, D_{t,i}\}_{T \geq t \geq 0}]$. The task is to sample the posterior distribution with probability density given by Bayes' Theorem,

$$p(\theta \mid \mathcal{D}_i) = \frac{p(\mathcal{D}_i \mid \theta) p(\theta)}{p(\mathcal{D}_i)},$$

where $p(\theta)$ is the prior, $p(\mathcal{D}_i \mid \theta)$ is the likelihood and $p(\mathcal{D}_i)$ is the evidence. For the remainder of this section, we omit the country index i for notational convenience.

B.1 Approximate Bayesian computation

For our model, the likelihood function can be written in terms of the solution to the forwards Kolmogorov equation, however, this requires large matrix exponentials to evaluate. Furthermore, since the full model state vector is only partially observable, the data model is no longer Markovian and is therefore computationally intractable. To deal with this likelihood intractability, we apply approximate Bayesian computation (ABC) [8–10], that samples from an approximation to the posterior for each country,

$$\begin{aligned} p(\theta \mid \mathcal{D}) \approx p(\theta \mid \rho(\mathcal{D}, \mathcal{D}_s) \leq \epsilon) &\propto \mathbb{P}(\rho(\mathcal{D}, \mathcal{D}_s) \leq \epsilon \mid \theta) p(\theta) \\ &= p(\theta) \int \mathbb{1}_{(0,\epsilon]} \left(\rho(\mathcal{D}, \mathcal{D}_s)\right) s(\mathcal{D}_s \mid \theta) \, \mathrm{d}\mathcal{D}_s, \end{aligned}$$

where $\mathcal{D}$ is the COVID-19 data for the country of interest, $\mathcal{D}_s \sim s(\cdot \mid \theta)$ is simulated data, $\rho(\mathcal{D}, \mathcal{D}_s)$ is a discrepancy metric, ϵ is the discrepancy threshold and $\mathbb{1}_{(0,\epsilon]} (\rho(\mathcal{D}, \mathcal{D}_s)) = 1$ if $\rho(\mathcal{D}, \mathcal{D}_s) \leq \epsilon$, and $\mathbb{1}_{(0,\epsilon]} (\rho(\mathcal{D}, \mathcal{D}_s)) = 0$ otherwise. For our implementation, we apply the discrepancy metric,

$$\rho(\mathcal{D}, \mathcal{D}_s) = \left(\sum_{t=1}^{T_d} (A_t - A_{t,s})^2 + (R_t - R_{t,s})^2 + (D_t - D_{t,s})^2 \right)^{1/2}$$

where $\mathcal{D} = [\{A_t, R_t, D_t\}_{t \geq 0}]$ is the data and $\mathcal{D}_s = [\{A_{t,s}, R_{t,s}, D_{t,s}\}_{t \geq 0}]$ is simulated data.

B.2 Sequential Monte Carlo sampling

We apply a sequential Monte Carlo (SMC) scheme [1,7] to move an initial set of N_p samples from the prior through a sequence of ABC approximations defined by a decreasing sequence of T discrepancy thresholds, $\epsilon_1 > \epsilon_2 > \cdots > \epsilon_T = \epsilon$. Our particular implementation (Algorithm 2), based on the work of Drovandi and Pettit [2], adaptively selects the acceptance thresholds and utilises MCMC steps using tuned Gaussian random walk proposals. For all model calibrations we apply adaptive SMC with $N_p = 2000$ particles, tuning parameters $c = 0.01$ and terminate sampling when the MCMC acceptance probably p_{acc} drops below $p_{\min} = 0.001$.

Algorithm 2 Adaptive SMC sampler for approxmate Bayesian computation

Initialise $N_a = aN_p$, $N_\ell = N_p - N_a$
for $j \in [1, 2, \ldots, N_p]$ **do**
  Sample prior, $\theta^* \sim p(\cdot)$ and simulate model, $\mathcal{D}_s \sim s(\cdot \mid \theta^*)$;
  Set $\rho_j \leftarrow \rho(\mathcal{D}, \mathcal{D}_s)$;
end for
repeat
  Sort particles $\{(\theta_j, \rho_j)\}_{j=1}^{N_p}$, such that $\rho_j \leq \rho_{j+1}$ for all $j \in [1, 2, \ldots, N_p - 1]$;
  Remove particles $\{(\theta_j, \rho_j)\}_{j=N_\ell+1}^{N_p}$ an set $\epsilon \leftarrow \rho_{N_\ell}$;
  Resample particles $\{\theta_j\}_{j=N_\ell+1}^{N_p}$ from $\{(\theta_j)\}_{j=1}^{N_\ell}$ with replacement;
  Estimate sample covariance, $\hat{\Sigma}$, of particles $\{\theta_j\}_{j=1}^{N_p}$.
  Adapt proposal kernel $q(u \mid v) = \phi\left(u; v, \dfrac{2.38}{\dim(\boldsymbol{\theta})}\hat{\Sigma}\right)$, where $\phi(\cdot; \mu, \Sigma)$ is a multivariate Gaussian density function and $\dim(\boldsymbol{\theta})$ is the number of parameters;
  Set $p_{\text{acc}} \leftarrow 0$;
  for $j \in [N_\ell + 1, N_\ell + 2, \ldots, N_p]$ **do**
    for $k \in [1, 2, \ldots, R_{\text{trial}}]$ **do**
      Generate proposal, $\theta^* \sim q(\cdot \mid \theta_j)$ and sample $u \sim \mathcal{U}(0, 1)$;
      if $u \leq \min\left(1, \dfrac{p(\theta^*)q(\theta_j \mid \theta^*)}{p(\theta_j)q(\theta^* \mid \theta_j)}\right)$ **then**
        Simulate model $\mathcal{D}_s \sim s(\cdot \mid \theta^*)$;
        if $\rho(\mathcal{D}_i, \mathcal{D}_s) \leq \epsilon$ **then**
          Set $\theta_j \leftarrow \theta^*$, $\rho_j \leftarrow \rho(\mathcal{D}_i, \mathcal{D}_s)$, and $p_{\text{acc}} \leftarrow p_{\text{acc}} + (R_{trial}N_a)^{-1}$;
        end if
      end if
    end for
  end for
  Set $R \leftarrow \log c / \log(1 - p_{\text{acc}})$;
  for $j \in [N_\ell + 1, N_\ell + 2, \ldots, N_p]$ **do**
    for $k \in [1, 2, \ldots, R - R_{\text{trial}}]$ **do**
      Generate proposal, $\theta^* \sim q(\cdot \mid \theta_j)$ and sample $u \sim \mathcal{U}(0, 1)$;
      if $u \leq \min\left(1, \dfrac{p(\theta^*)q(\theta_j \mid \theta^*)}{p(\theta_j)q(\theta^* \mid \theta_j)}\right)$ **then**
        Simulate model $\mathcal{D}_s \sim s(\cdot \mid \theta^*)$;
        if $\rho(\mathcal{D}_i, \mathcal{D}_s) \leq \epsilon$ **then**
          Set $\theta_j \leftarrow \theta^*$, $\rho_j \leftarrow \rho(\mathcal{D}_i, \mathcal{D}_s)$, and $p_{\text{acc}} \leftarrow p_{\text{acc}} + (RN_a)^{-1}$;
        end if
      end if
    end for
  end for
until $p_{\text{acc}} < p_{\min}$

B.3 Point estimate computation

Given a set of particles, $\{\theta_j\}_{j=1}^{N_p}$, from the ABC posterior generated from the SMC sampler, we require a point estimate for each parameter to compare overall trends between countries. We found the posterior means often did not result is a posterior predictive distribution that was representative of the data for most countries.

Instead we take the ABC posterior particle that has the smallest average discrepancy with the data. That is, for country i, the point estimate as

$$\hat{\theta}_i = \underset{\theta \in \{\theta_j\}_{j=1}^{N_p}}{\operatorname{argmin}} \int \rho(\mathcal{D}_i, \mathcal{D}_s) s(\mathcal{D}_s \mid \theta)\, \mathrm{d}\mathcal{D}_s.$$

We evaluate this through direct Monte Carlo (Algorithm 3) using $M = 100$ simulations per particle.

Algorithm 3 Monte Carlo method for computing point estimates

```
Give data $\mathcal{D}_i$ and ABC posterior samples $\{\theta_j\}_{j=1}^{N_p}$ generated by SMC (Algorithm 2);
Set $\bar{\rho}_{\min} \leftarrow \infty$;
for $j \in \{1, 2, \dots, N_p\}$ do
    Generate simulations $\mathcal{D}_s^{(1)}, \mathcal{D}_s^{(2)}, \dots, \mathcal{D}_s^{(M)} \sim s(\mathcal{D}_s \mid \theta_j)$;
    Set $\bar{\rho} \leftarrow \frac{1}{M}\sum_{k=1}^{M} \rho(\mathcal{D}_i, \mathcal{D}_s^{(k)})$;
    if $\bar{\rho} < \bar{\rho}_{\min}$ then
        Set $\bar{\rho}_{\min} \leftarrow \bar{\rho}$ and $\hat{\theta}_i \leftarrow \theta_j$
    end if
end for
```

Appendix C Marginal posterior comparisons

This section includes box-plots for comparison of marginal posterior distributions across countries for the model parameters $\theta = [\alpha_0, \alpha, \beta, \gamma, \eta, n, \kappa, w_A]$. Results for the first analysis period (22 January–30 March) are given the Fig. 1–3, results for the second analysis period (22 January–13 April) are given the Fig. 4–6, and results for the third analysis period (22 January–9 June) are given the Fig. 7–9. For reference, Table 1 provides the ISO-3166 alpha3 codes for each country.

Table 1: Lookup table of Country names by ISO-3166 alpha3 codes

Country Code	Country Name	Country Code	Country Name
AFG	Afghanistan	JPN	Japan
ALB	Albania	KAZ	Kazakhstan
AND	Andorra	KHM	Cambodia
ARE	United Arab Emirates	KOR	South Korea
ARG	Argentina	KWT	Kuwait
ARM	Armenia	LBN	Lebanon
AUS	Australia	LKA	Sri Lanka
AUT	Austria	LTU	Lithuania
AZE	Azerbaijan	LUX	Luxembourg
BEL	Belgium	LVA	Latvia
BFA	Burkina Faso	MAR	Morocco
BGR	Bangladesh	MDA	Moldova
BHR	Bahrain	MEX	Mexico
BIH	Bosnia and Herzegovina	MKD	North Macedonia
BRA	Brazil	MLT	Malta
BRN	Brunei	MUS	Mauritius
CAN	Canada	MYS	Malaysia
CHE	Switzerland	NGA	Nigeria
CHL	Chile	NLD	Netherlands
CHN	China	NOR	Norway
CIV	Cote d'Ivoire	NZL	New Zealand
CMR	Camaroon	OMN	Oman
COL	Columbia	PAK	Pakistan
CRI	Costa Rica	PAN	Panama
CUB	Cuba	PER	Peru
CYP	Cyprus	PHL	Philippines
CZE	Czechia	POL	Poland
DEU	Germany	PRT	Portugal
DNK	Denmark	PSE	Palestine
DOM	Dominican Republic	QAT	Qatar
DZA	Algeria	ROU	Romania
ECU	Ecuador	RUS	Russia
EGY	Egypt	SAU	Saudi Arabia
ESP	Spain	SEN	Senegal
EST	Estonia	SGP	Singapore
FIN	Finland	SMR	San Marino
FRA	France	SRB	Serbia
GBR	United Kingdom	SVK	Slovakia
GHA	Ghana	SVN	Slovenia
GRC	Greece	SWE	Sweden
HND	Honduras	THA	Thailand
HRV	Croatia	TUN	Tunisia
HUN	Hungary	TUR	Turkey
IDN	Indonesia	TWN	Taiwan
IND	India	UKR	Ukraine
IRL	Republic of Ireland	URY	Uruguay
IRN	Iran	USA	United States
IRQ	Iraq	UZB	Uzbekistan
ISL	Iceland	VEN	Venezuela
ISR	Israel	VNM	Vietnam
ITA	Italy	ZAF	South Africa
JOR	Jordan		

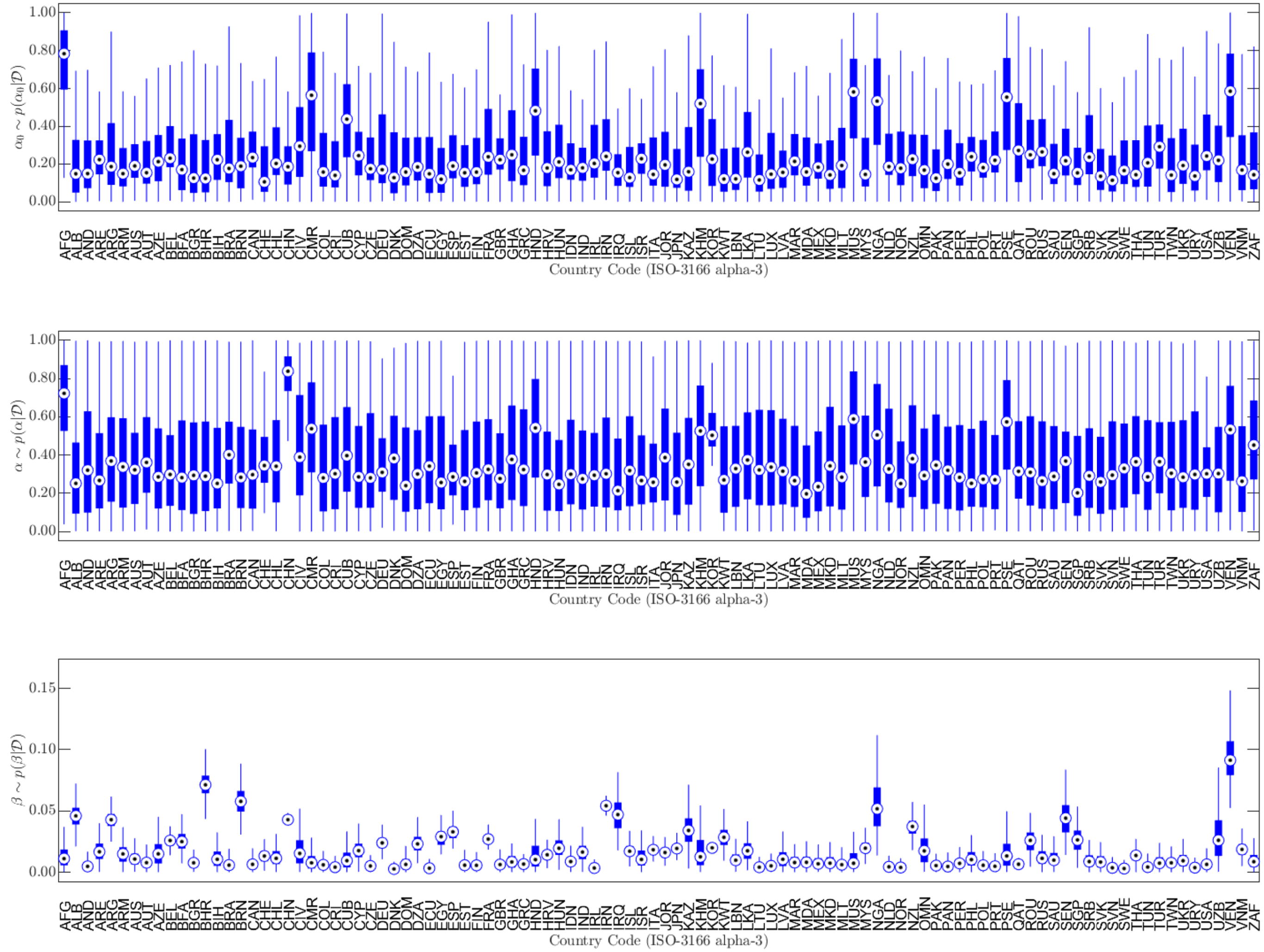

Figure 1: Box plots comparing marginal posterior distributions by country over the period 22 January–30 March 2020 for parameters α_0 (top), α, and β (bottom).

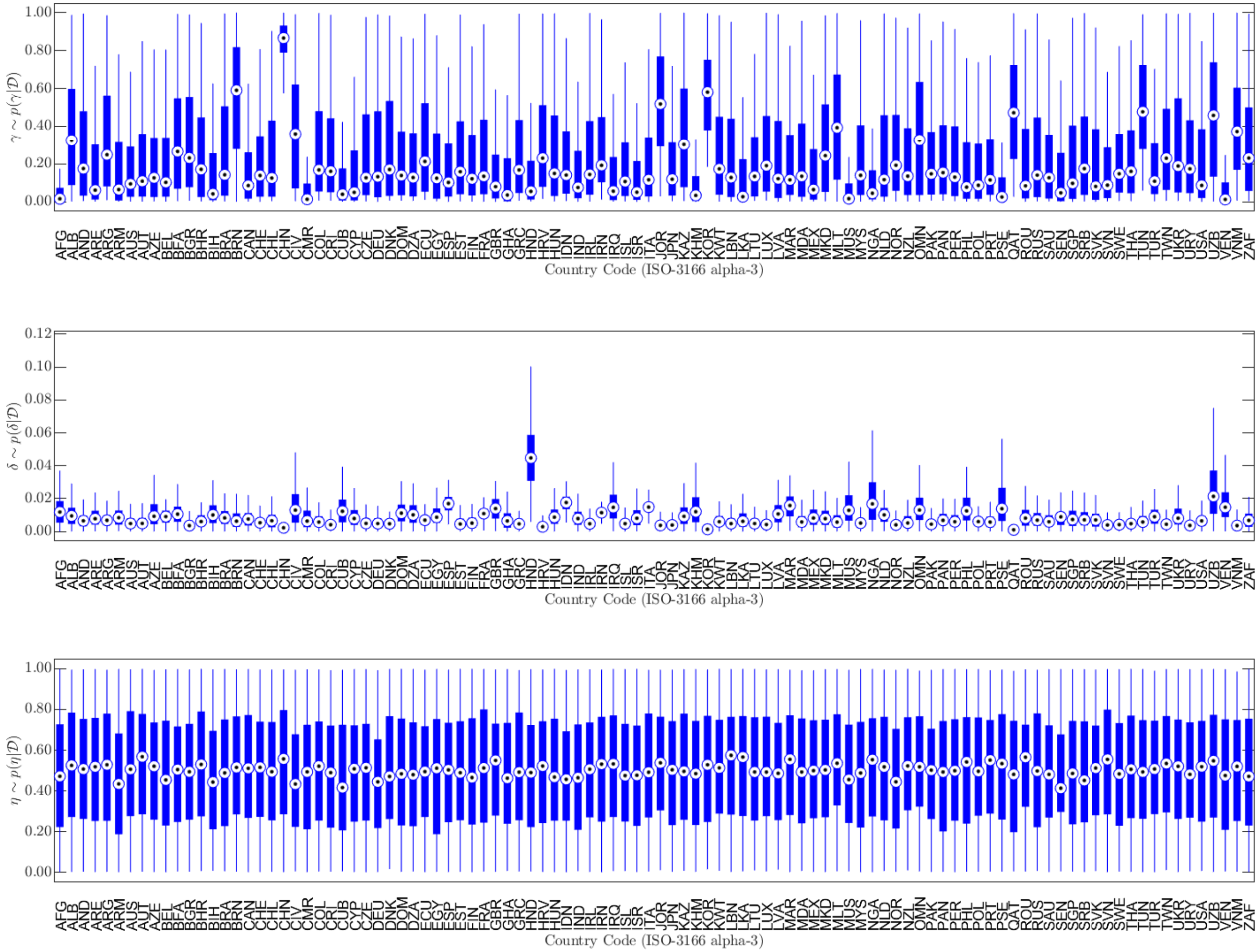

Figure 2: Box plots comparing marginal posterior distributions by country over the period 22 January–30 March 2020 for parameters γ (top), δ, and η (bottom).

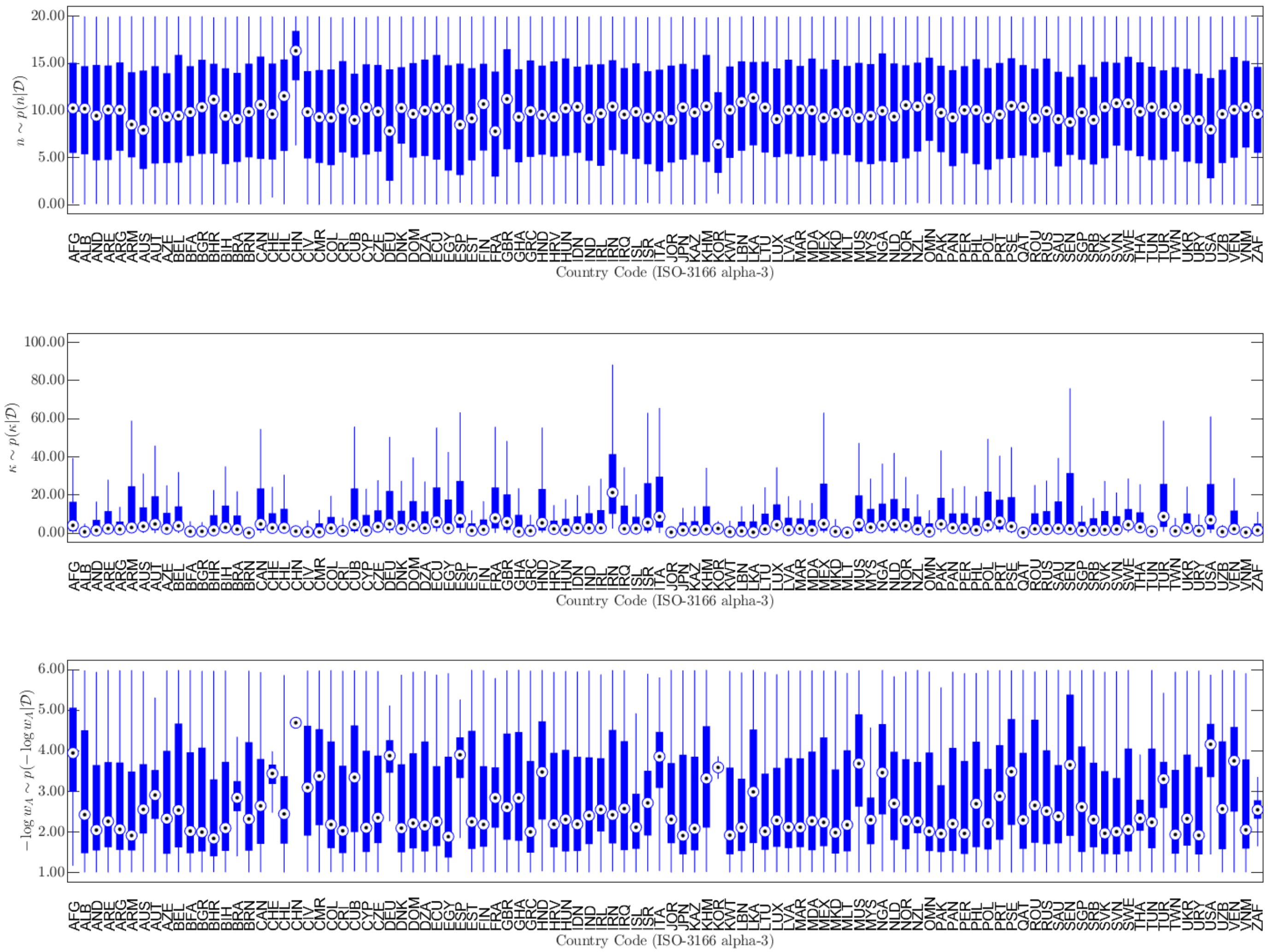

Figure 3: Box plots comparing marginal posterior distributions by country over the period 22 January–30 March 2020 for parameters n (top), κ, and $-\log_{10} w_A$ (bottom).

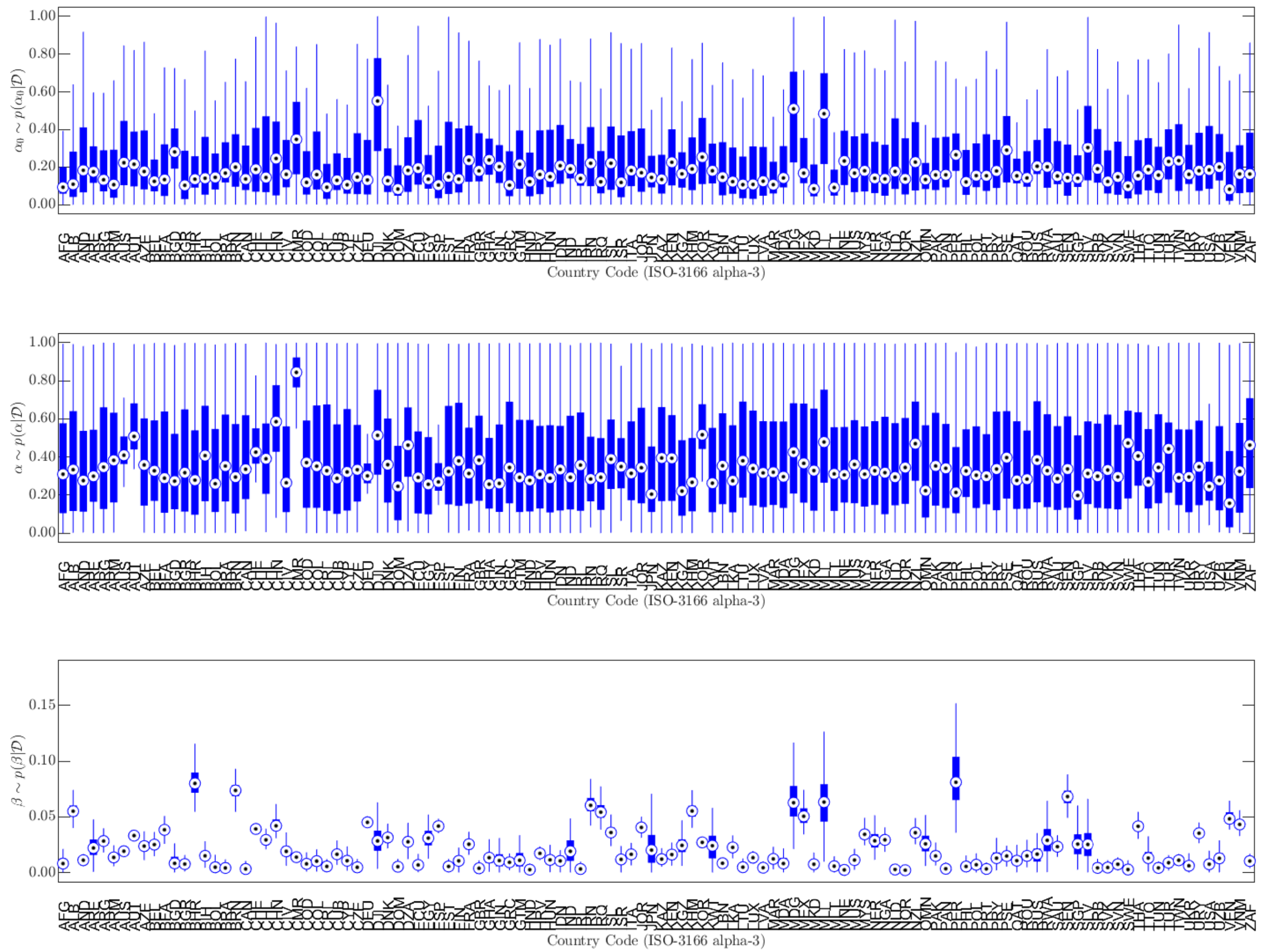

Figure 4: Box plots comparing marginal posterior distributions by country over the period 22 January–13 April 2020 for parameters α_0 (top), α, and β (bottom).

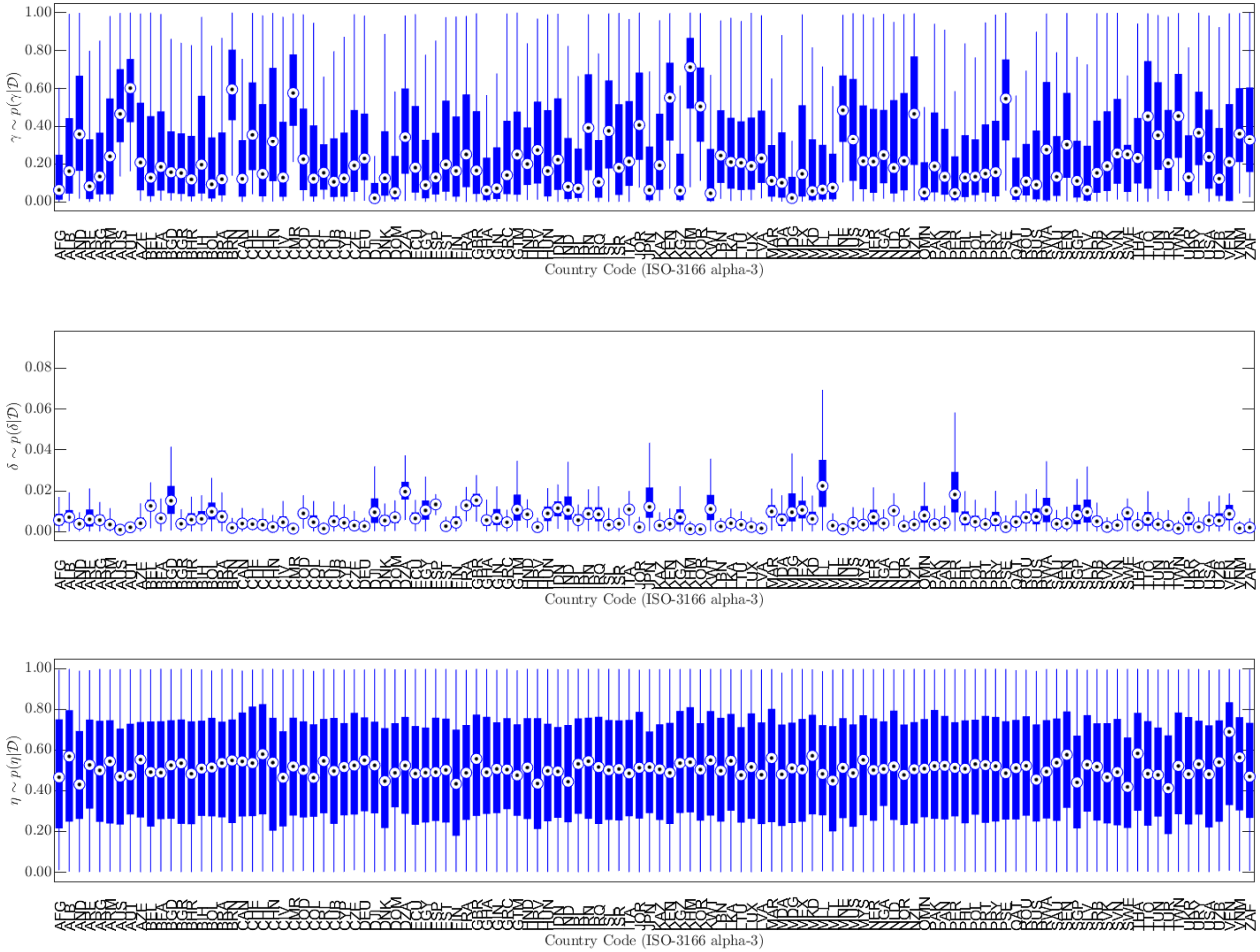

Figure 5: Box plots comparing marginal posterior distributions by country over the period 22 January–13 April 2020 for parameters γ (top), δ, and η (bottom).

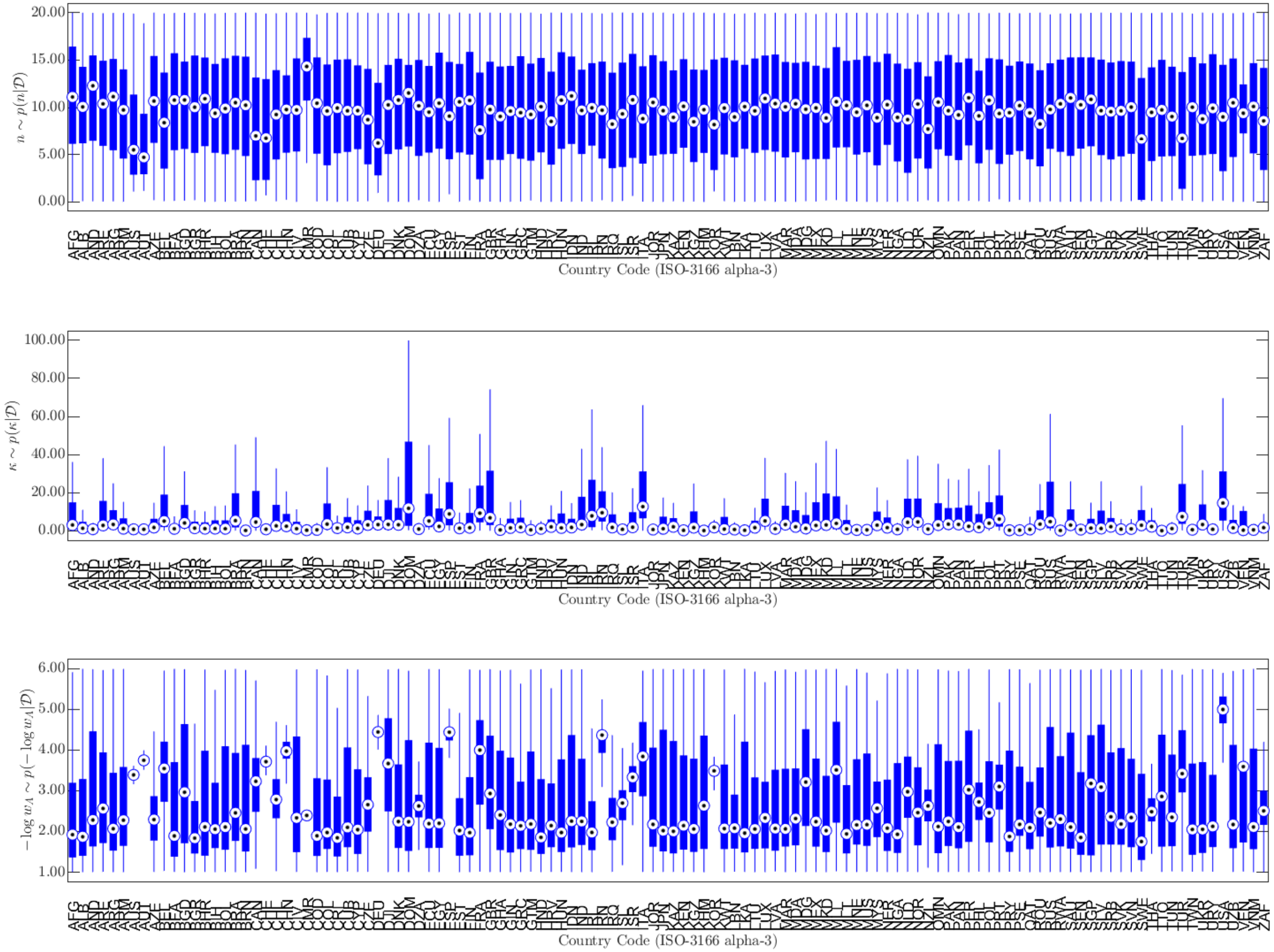

Figure 6: Box plots comparing marginal posterior distributions by country over the period 22 January–13 April 2020 for parameters n (top), κ, and $-\log_{10} w_A$ (bottom).

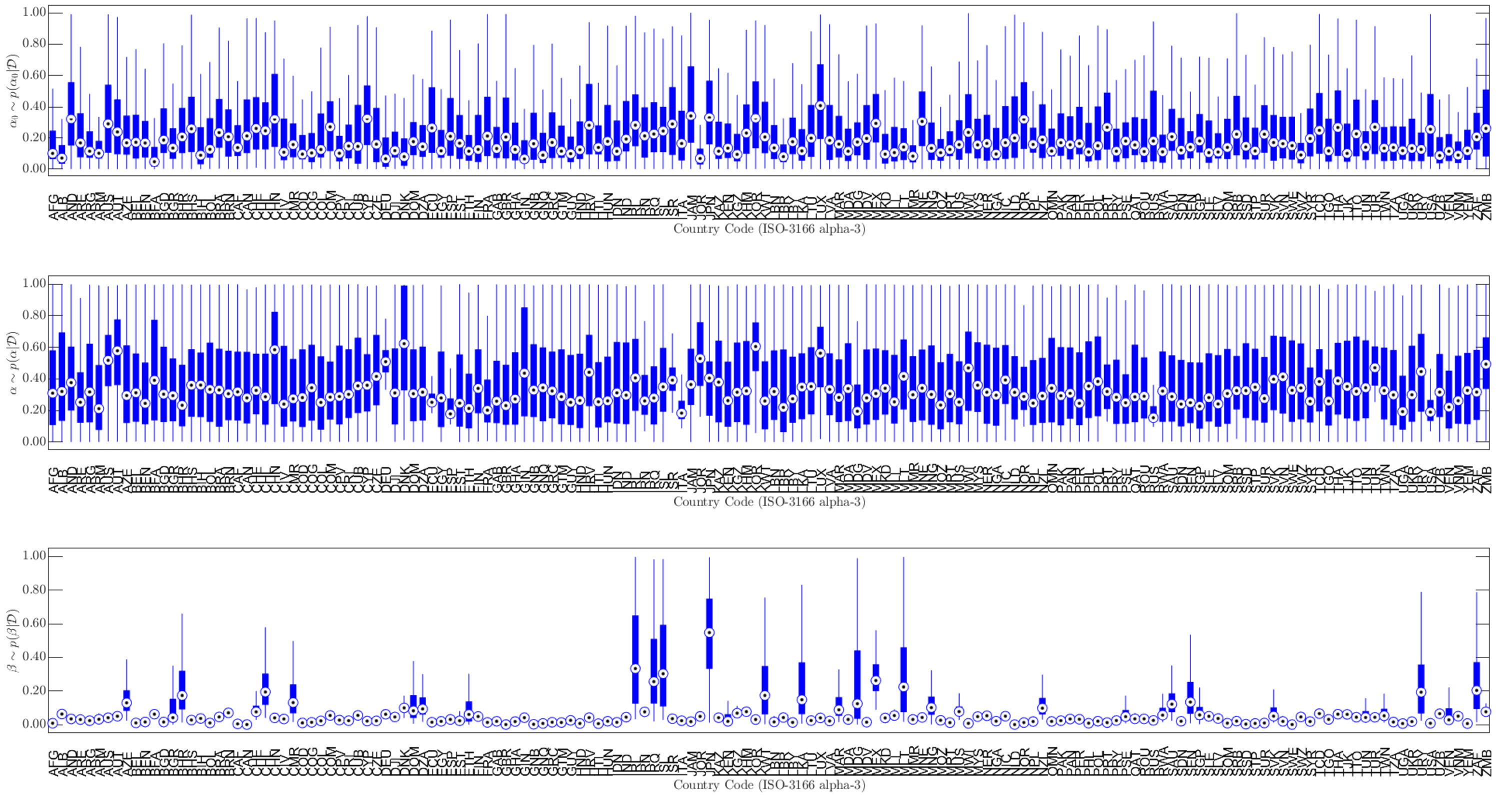

Figure 7: Box plots comparing marginal posterior distributions by country over the period 22 January–9 June 2020 for parameters α_0 (top), α, and β (bottom).

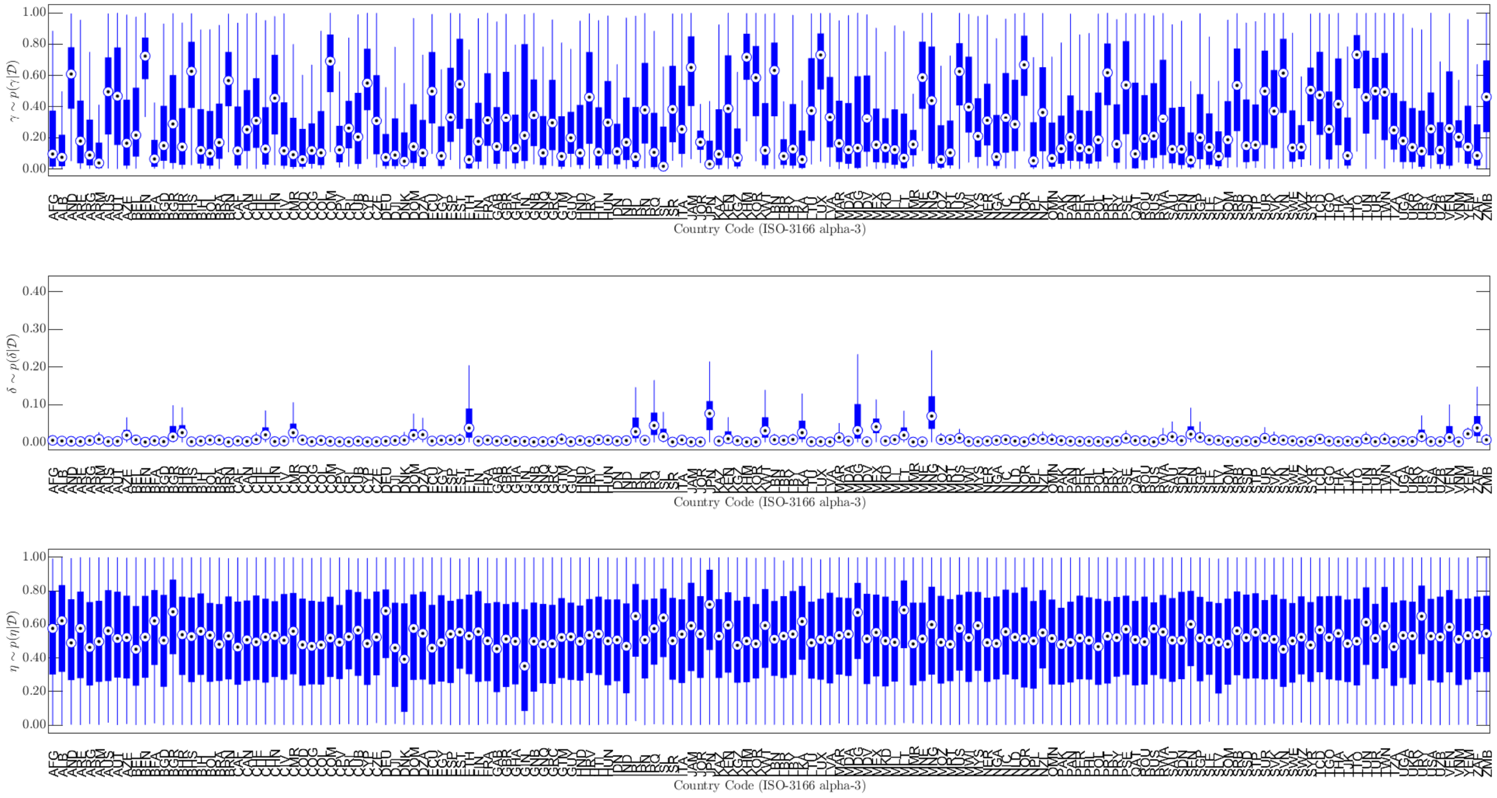

Figure 8: Box plots comparing marginal posterior distributions by country over the period 22 January–9 June 2020 for parameters γ (top), δ, and η (bottom).

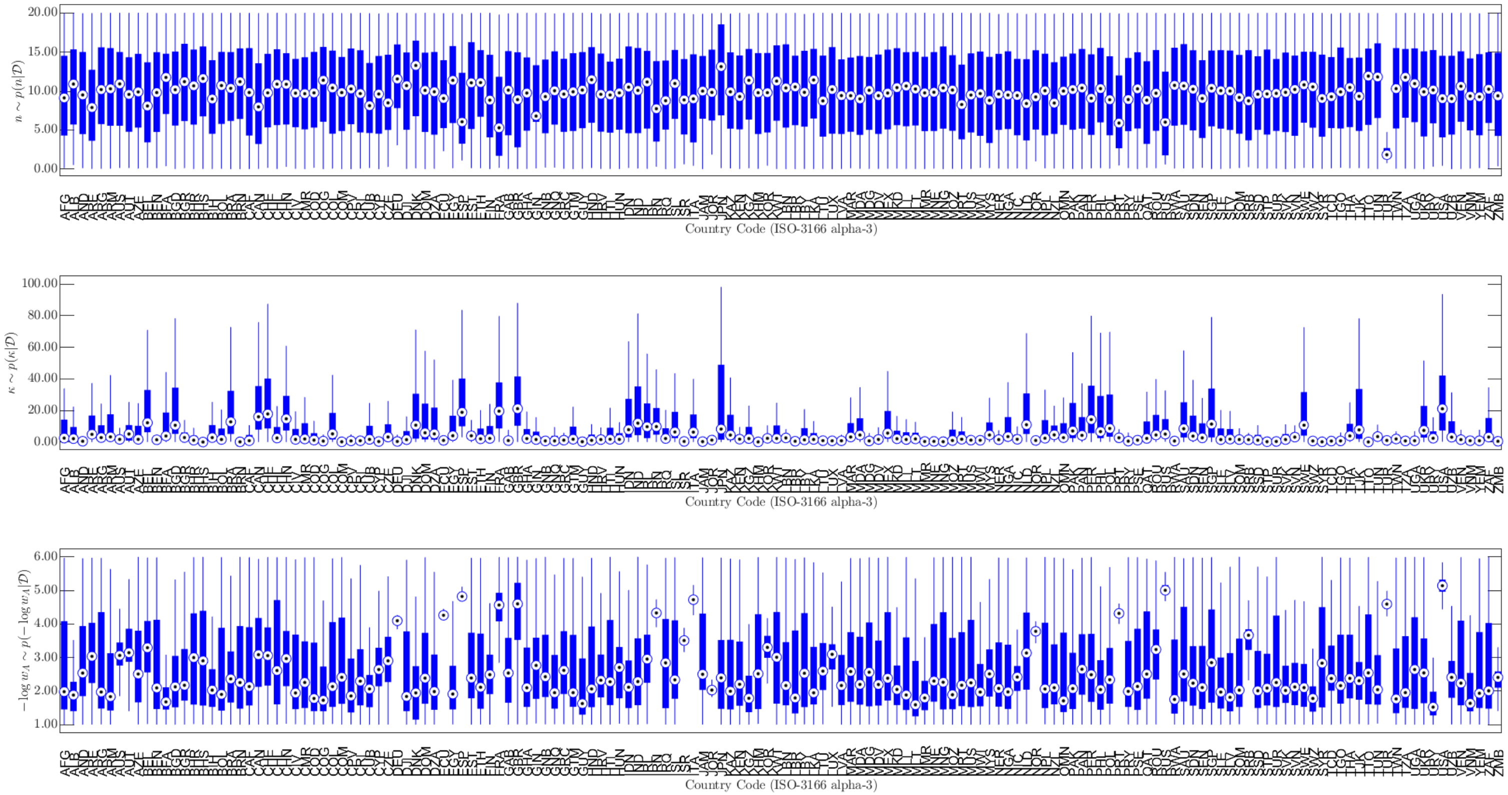

Figure 9: Box plots comparing marginal posterior distributions by country over the period 22 January–9 June 2020 for parameters n (top), κ, and $-\log_{10} w_A$ (bottom).

Appendix D Example posterior predictive distributions

Here we present examples posterior predictive distributions. In general, our model tends to smooth over sudden spikes in daily reported cases, recoveries and fatalities.

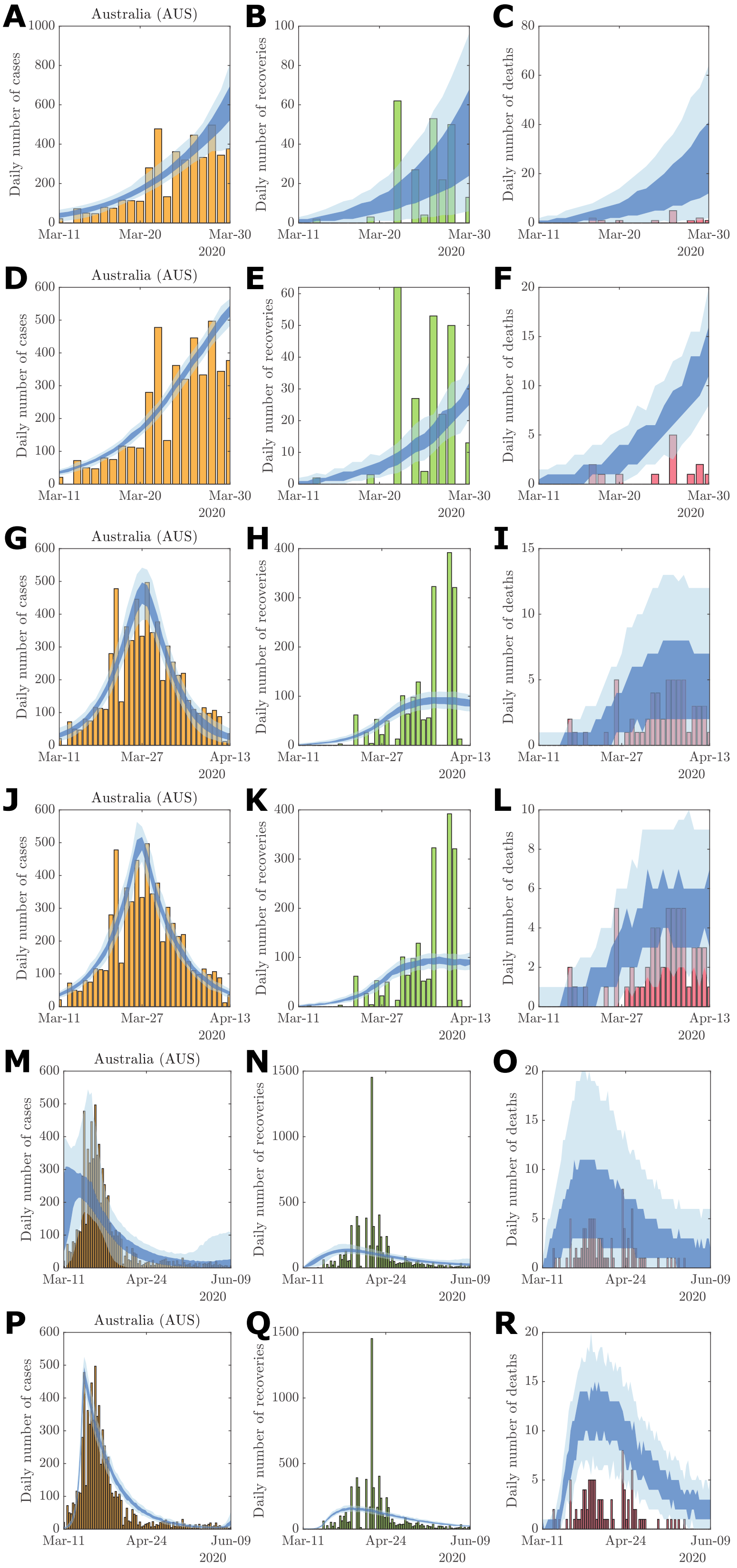

Figure 10: Full posterior predictive distributions for Australia over the periods (A)–(C) 22 January–30 March, (G)–(I) 22 January–13 April, and (M)–(O) 22 January–9 June, are compared with model fit using the parameter point estimate for periods (D)–(F) 22 January–30 March, (J)–(L) 22 January–13 April, and (P)–(R) 22 January–9 June. Vertical bars indicate daily reported cases (yellow), recoveries (green) and deaths (red). The 50% (dark shaded region) and 95% credible intervals (light shaded region) of the posterior predictive distributions are plotted against the observational data.

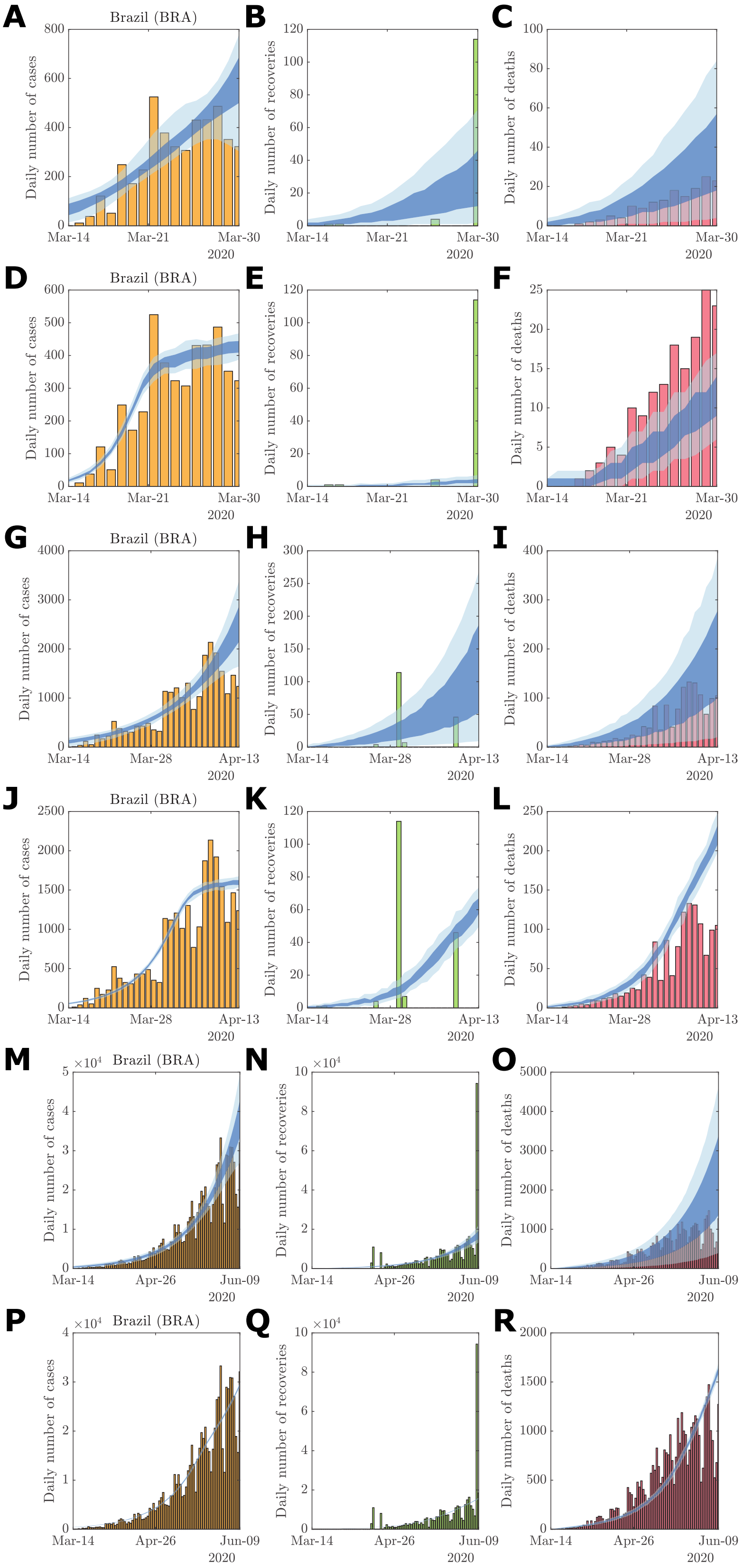

Figure 11: Full posterior predictive distributions for Brazil over the periods (A)–(C) 22 January–30 March, (G)–(I) 22 January–13 April, and (M)–(O) 22 January–9 June, are compared with model fit using the parameter point estimate for periods (D)–(F) 22 January–30 March, (J)–(L) 22 January–13 April, and (P)–(R) 22 January–9 June. Vertical bars indicate daily reported cases (yellow), recoveries (green) and deaths (red). The 50% (dark shaded region) and 95% credible intervals (light shaded region) of the posterior predictive distributions are plotted against the observational data.

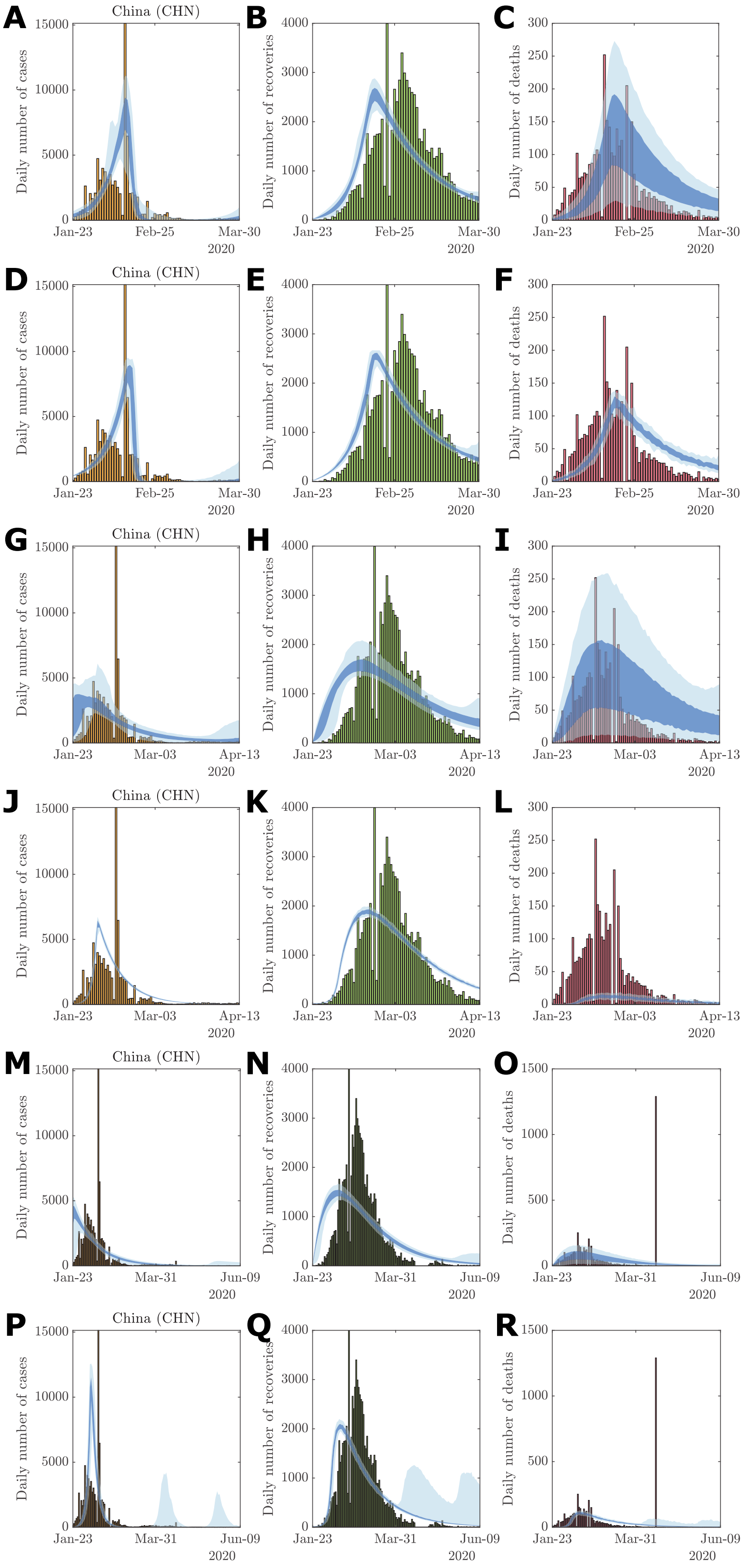

Figure 12: Full posterior predictive distributions for China over the periods (A)–(C) 22 January–30 March, (G)–(I) 22 January–13 April, and (M)–(O) 22 January–9 June, are compared with model fit using the parameter point estimate for periods (D)–(F) 22 January–30 March, (J)–(L) 22 January–13 April, and (P)–(R) 22 January–9 June. Vertical bars indicate daily reported cases (yellow), recoveries (green) and deaths (red). The 50% (dark shaded region) and 95% credible intervals (light shaded region) of the posterior predictive distributions are plotted against the observational data.

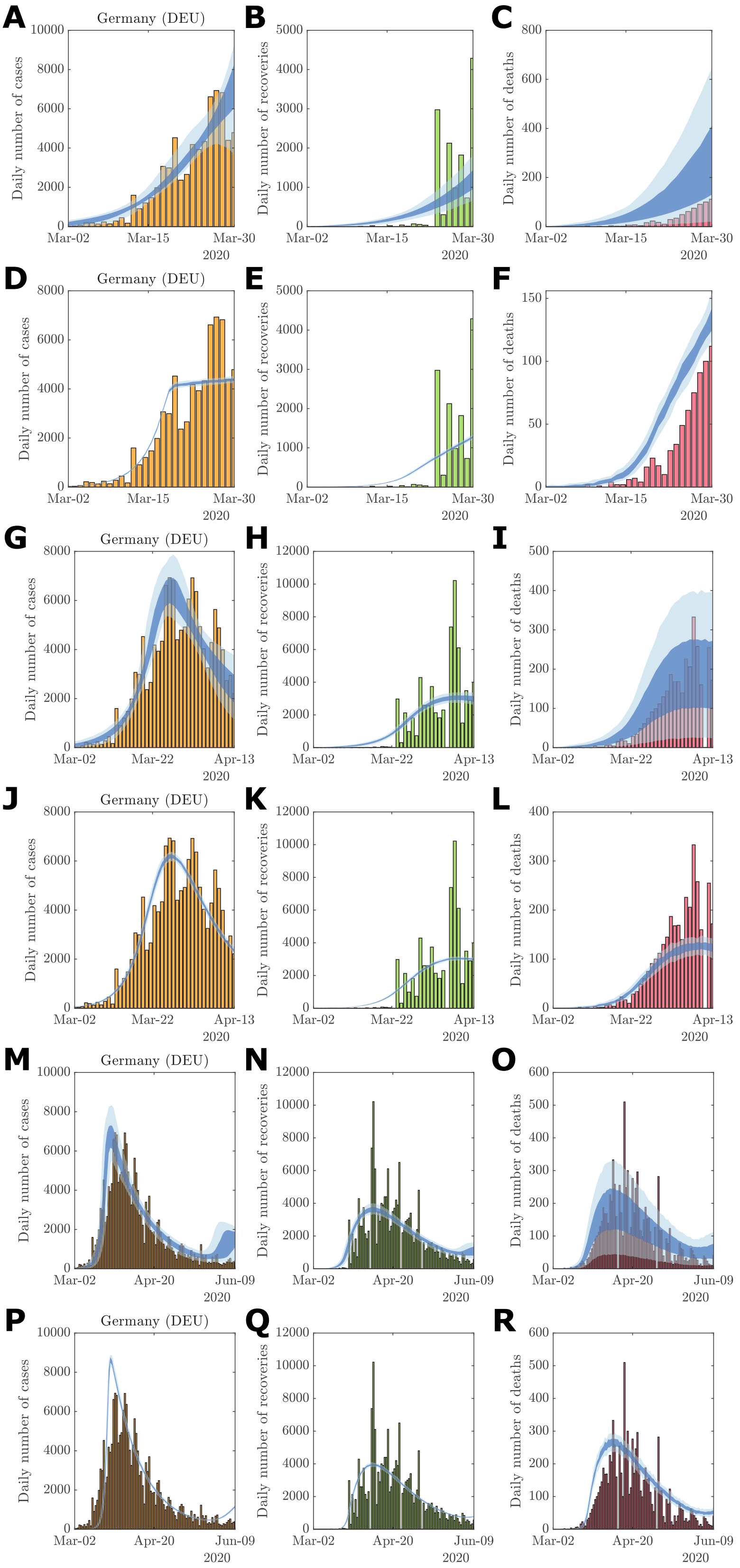

Figure 13: Full posterior predictive distributions for Germany over the periods (A)–(C) 22 January–30 March, (G)–(I) 22 January–13 April, and (M)–(O) 22 January–9 June, are compared with model fit using the parameter point estimate for periods (D)–(F) 22 January–30 March, (J)–(L) 22 January–13 April, and (P)–(R) 22 January–9 June. Vertical bars indicate daily reported cases (yellow), recoveries (green) and deaths (red). The 50% (dark shaded region) and 95% credible intervals (light shaded region) of the posterior predictive distributions are plotted against the observational data.

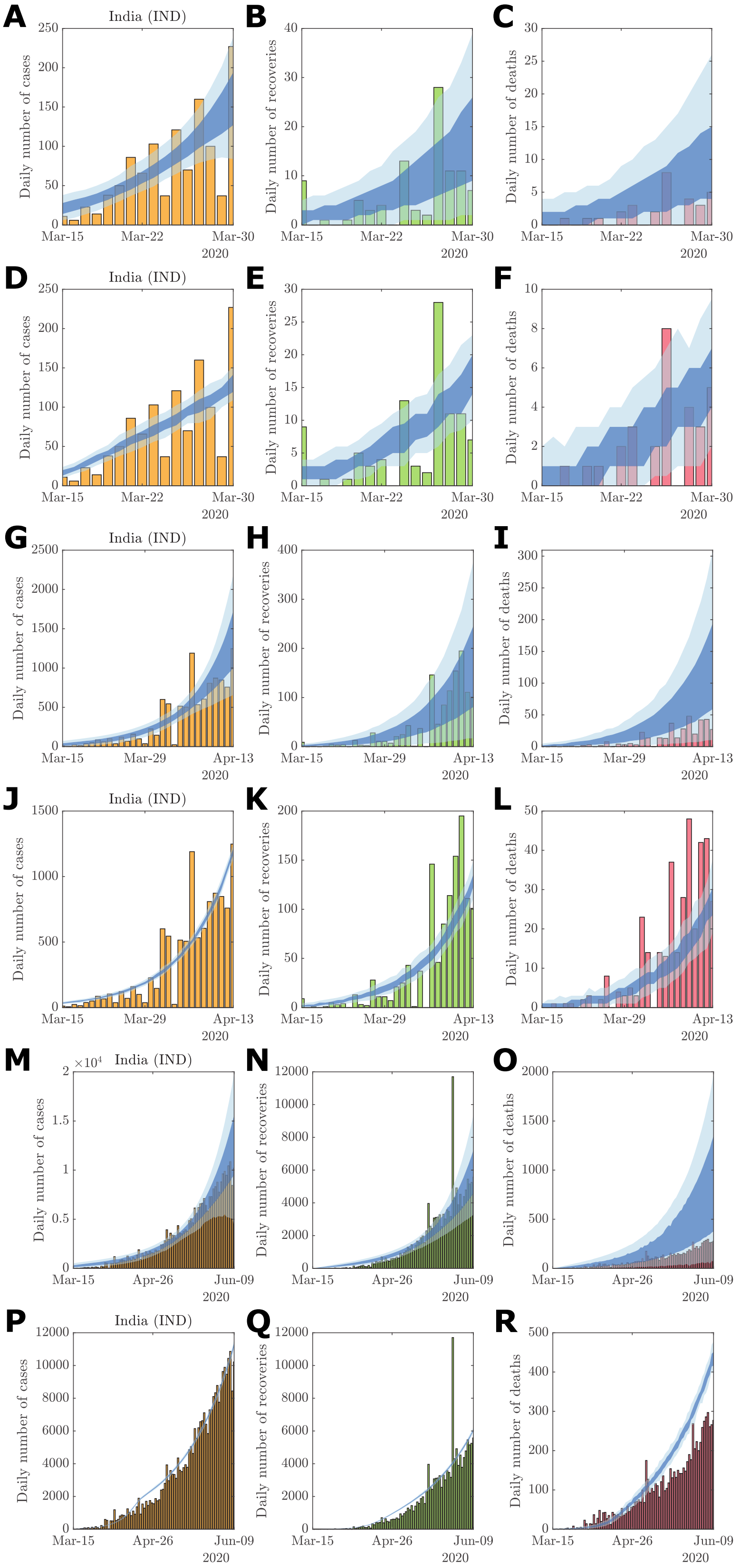

Figure 14: Full posterior predictive distributions for India over the periods (A)–(C) 22 January–30 March, (G)–(I) 22 January–13 April, and (M)–(O) 22 January–9 June, are compared with model fit using the parameter point estimate for periods (D)–(F) 22 January–30 March, (J)–(L) 22 January–13 April, and (P)–(R) 22 January–9 June. Vertical bars indicate daily reported cases (yellow), recoveries (green) and deaths (red). The 50% (dark shaded region) and 95% credible intervals (light shaded region) of the posterior predictive distributions are plotted against the observational data.

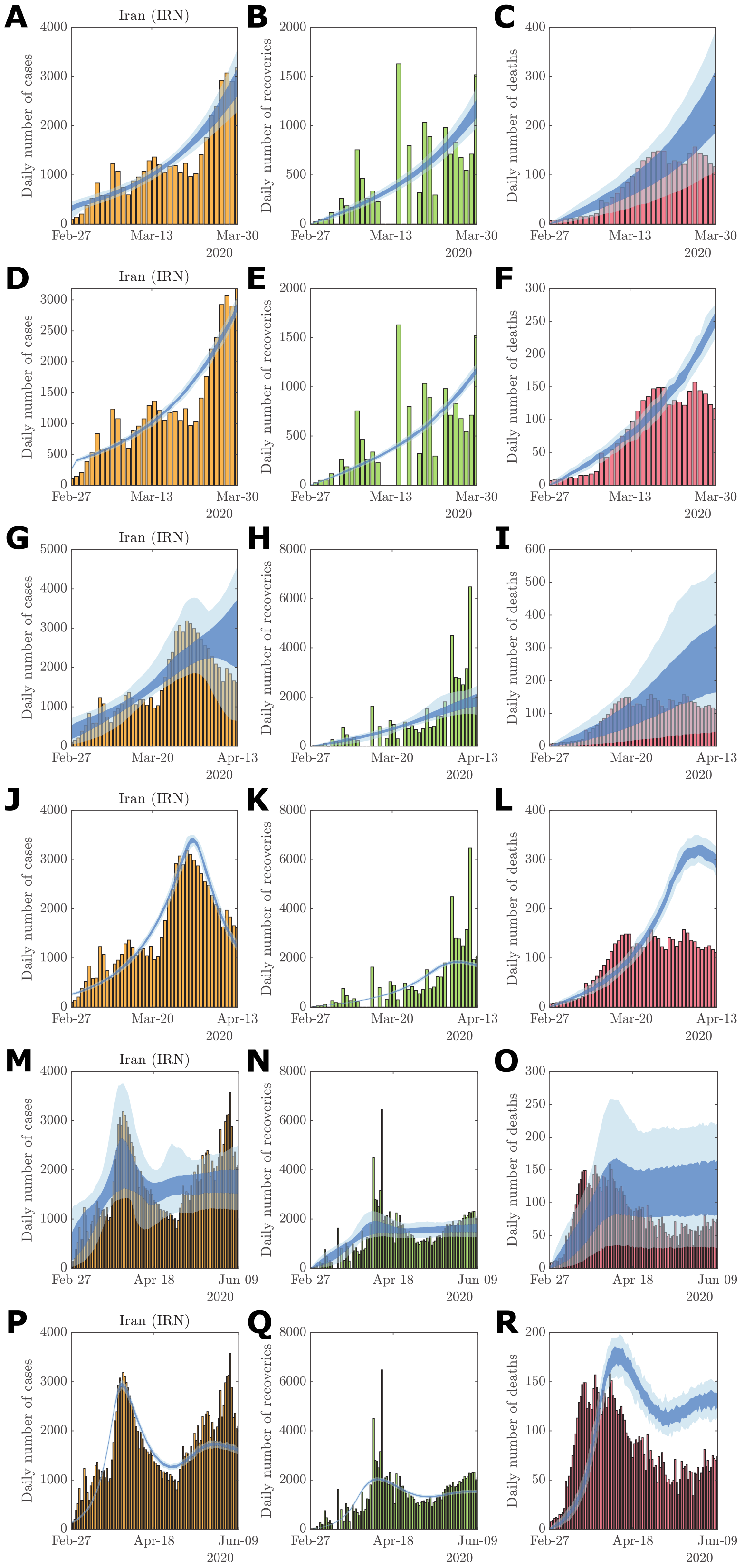

Figure 15: Full posterior predictive distributions for Iran over the periods (A)–(C) 22 January–30 March, (G)–(I) 22 January–13 April, and (M)–(O) 22 January–9 June, are compared with model fit using the parameter point estimate for periods (D)–(F) 22 January–30 March, (J)–(L) 22 January–13 April, and (P)–(R) 22 January–9 June. Vertical bars indicate daily reported cases (yellow), recoveries (green) and deaths (red). The 50% (dark shaded region) and 95% credible intervals (light shaded region) of the posterior predictive distributions are plotted against the observational data.

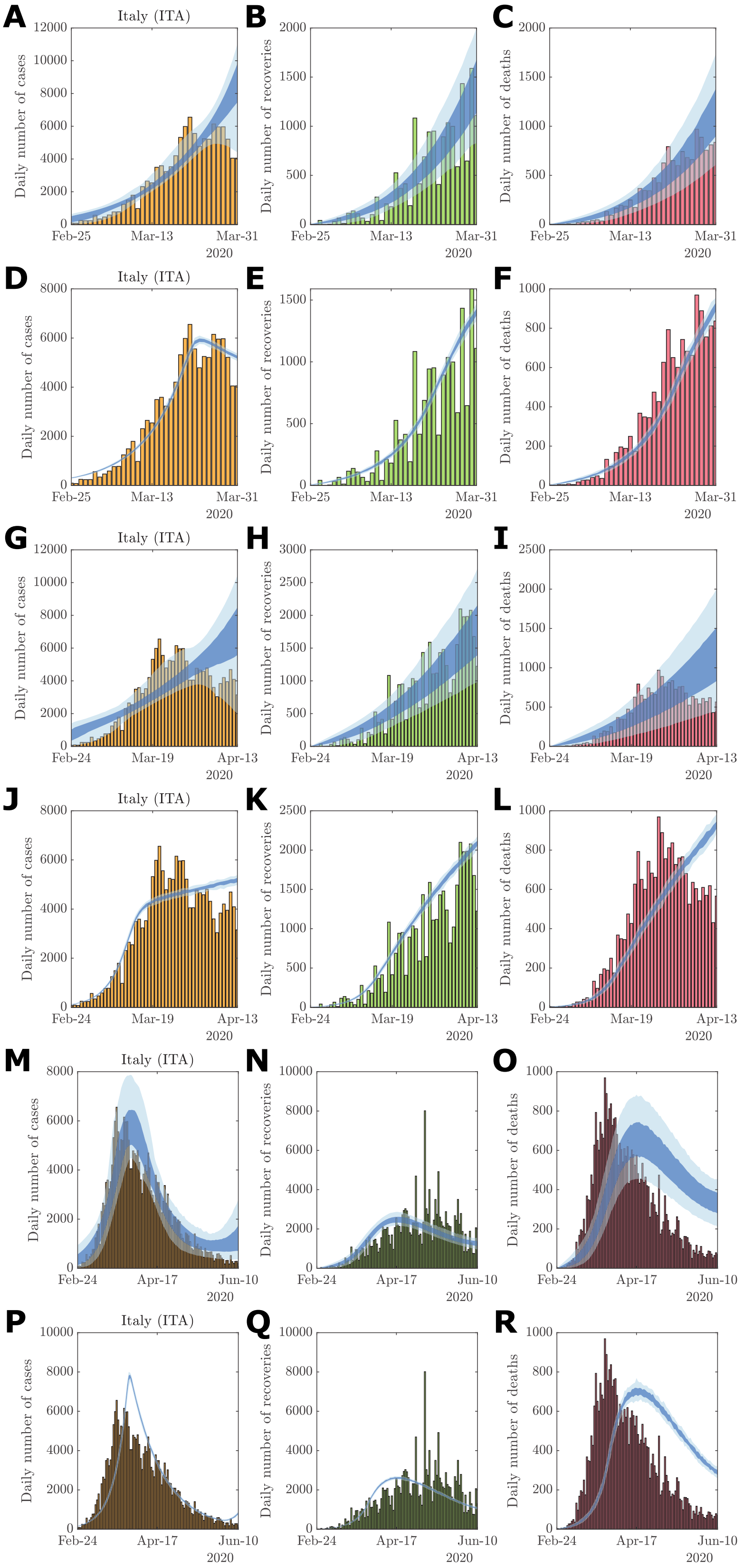

Figure 16: Full posterior predictive distributions for Italy over the periods (A)–(C) 22 January–30 March, (G)–(I) 22 January–13 April, and (M)–(O) 22 January–9 June, are compared with model fit using the parameter point estimate for periods (D)–(F) 22 January–30 March, (J)–(L) 22 January–13 April, and (P)–(R) 22 January–9 June. Vertical bars indicate daily reported cases (yellow), recoveries (green) and deaths (red). The 50% (dark shaded region) and 95% credible intervals (light shaded region) of the posterior predictive distributions are plotted against the observational data.

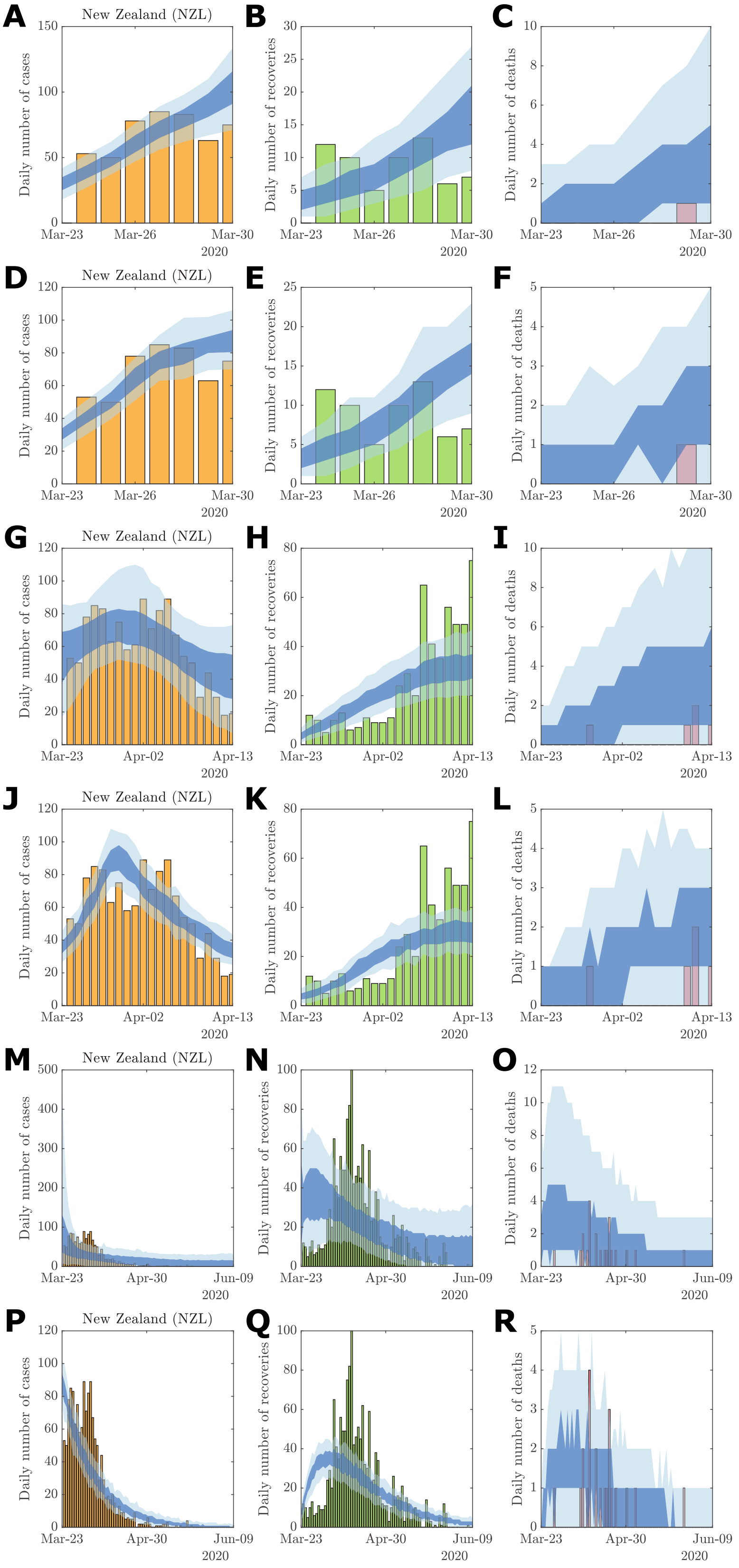

Figure 17: Full posterior predictive distributions for New Zealand over the periods (A)–(C) 22 January–30 March, (G)–(I) 22 January–13 April, and (M)–(O) 22 January–9 June, are compared with model fit using the parameter point estimate for periods (D)–(F) 22 January–30 March, (J)–(L) 22 January–13 April, and (P)–(R) 22 January–9 June. Vertical bars indicate daily reported cases (yellow), recoveries (green) and deaths (red). The 50% (dark shaded region) and 95% credible intervals (light shaded region) of the posterior predictive distributions are plotted against the observational data.

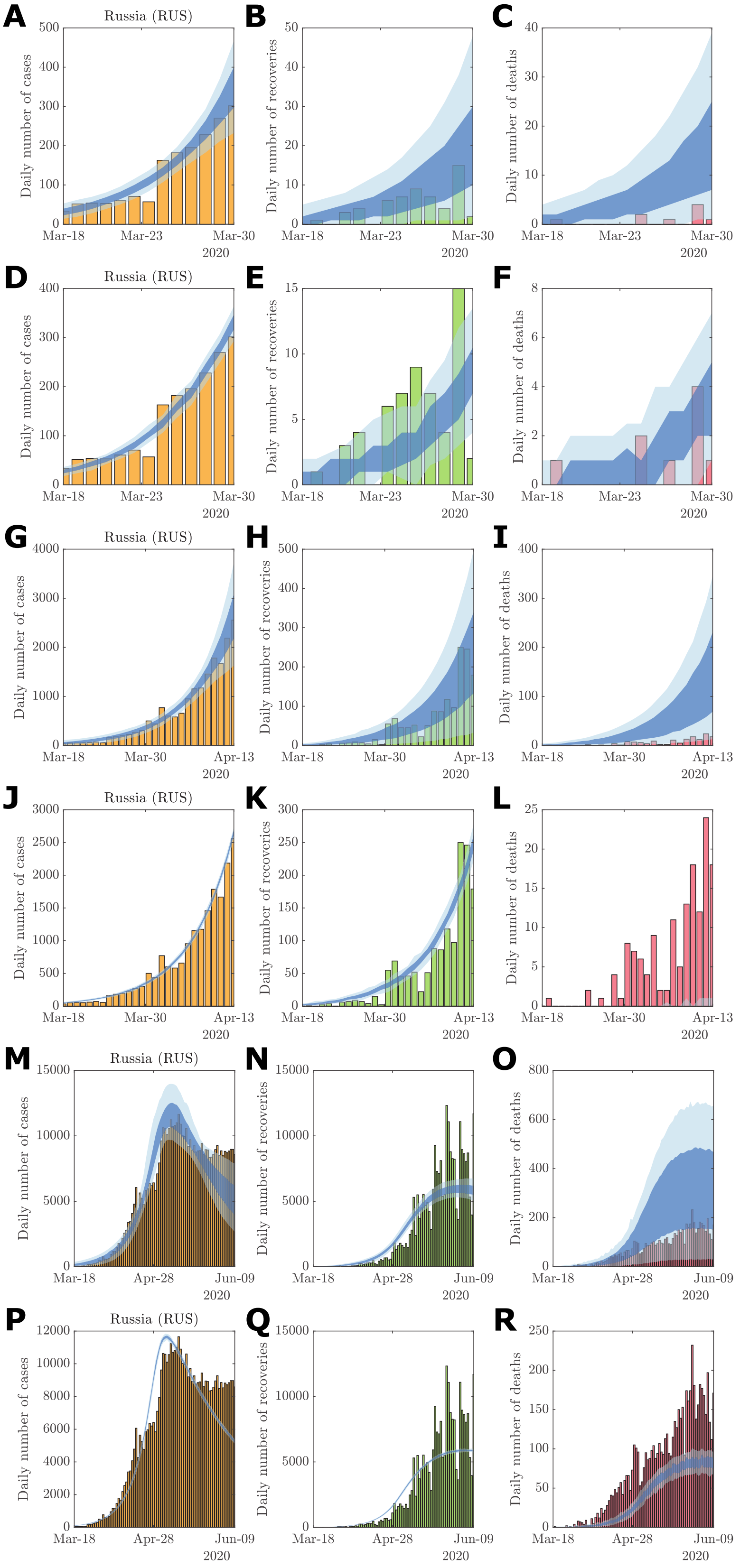

Figure 18: Full posterior predictive distributions for Russia over the periods (A)–(C) 22 January–30 March, (G)–(I) 22 January–13 April, and (M)–(O) 22 January–9 June, are compared with model fit using the parameter point estimate for periods (D)–(F) 22 January–30 March, (J)–(L) 22 January–13 April, and (P)–(R) 22 January–9 June. Vertical bars indicate daily reported cases (yellow), recoveries (green) and deaths (red). The 50% (dark shaded region) and 95% credible intervals (light shaded region) of the posterior predictive distributions are plotted against the observational data.

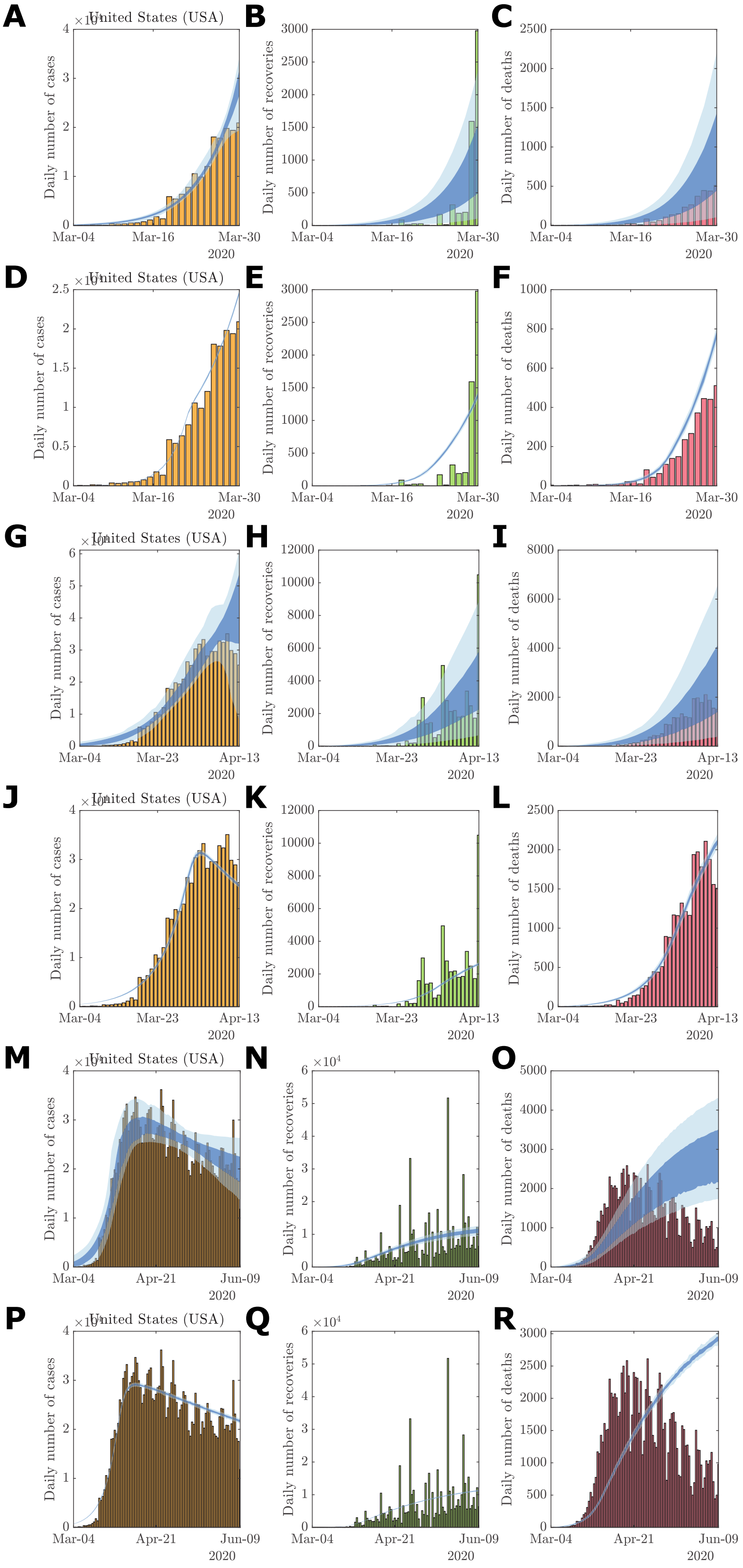

Figure 19: Full posterior predictive distributions for the United States over the periods (A)–(C) 22 January–30 March, (G)–(I) 22 January–13 April, and (M)–(O) 22 January–9 June, are compared with model fit using the parameter point estimate for periods (D)–(F) 22 January–30 March, (J)–(L) 22 January–13 April, and (P)–(R) 22 January–9 June. Vertical bars indicate daily reported cases (yellow), recoveries (green) and deaths (red). The 50% (dark shaded region) and 95% credible intervals (light shaded region) of the posterior predictive distributions are plotted against the observational data.

Appendix E Sensitivity analysis

We performed a sensitivity analysis of our approach using the full response function $U(A_t, R_t, D_t) = w_A A_t + w_R R_t + w_D D_t$ compared with our reduced model used in the analysis by setting $w_R = w_D = 0$. Overall, the resulting distribution of point estimates and correlation coefficients did not change substantially (Compare Fig. 20 with Fig. 21). We conclude that a community response that is based on active case numbers is a sufficient first order approximation for broad global comparison.

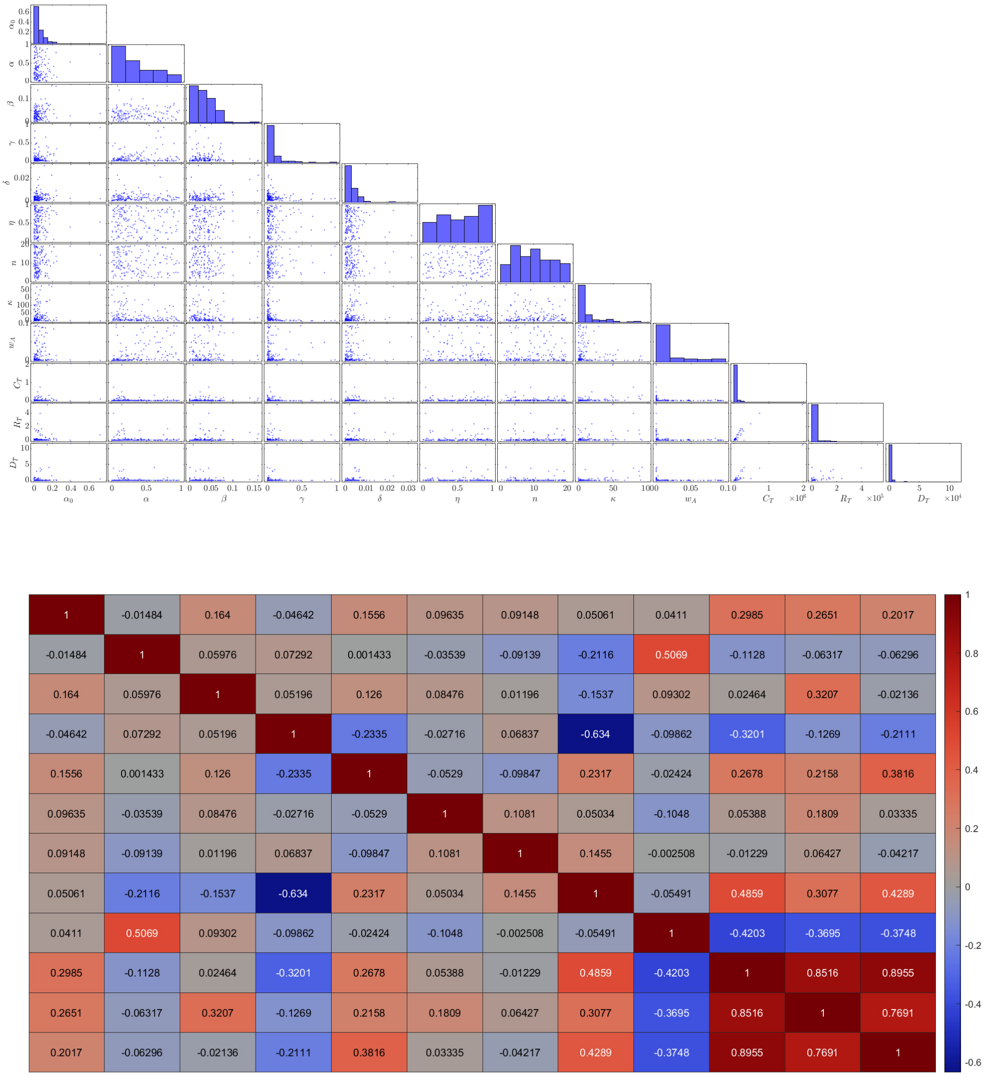

Figure 20: (top) Distributions of model parameter point using reduced model (that is $w_R = w_D = 0$) estimates along with observered cumulative confirmed cases C_T, recoveries R_T and deaths D_T at $T = 9$ June. (bottom) Spearman correlation coefficients between each point estimate and observed case numbers with the sign and strength of the correlation indicated by the colour-map (positive correlations in red and negative correlations in blue).

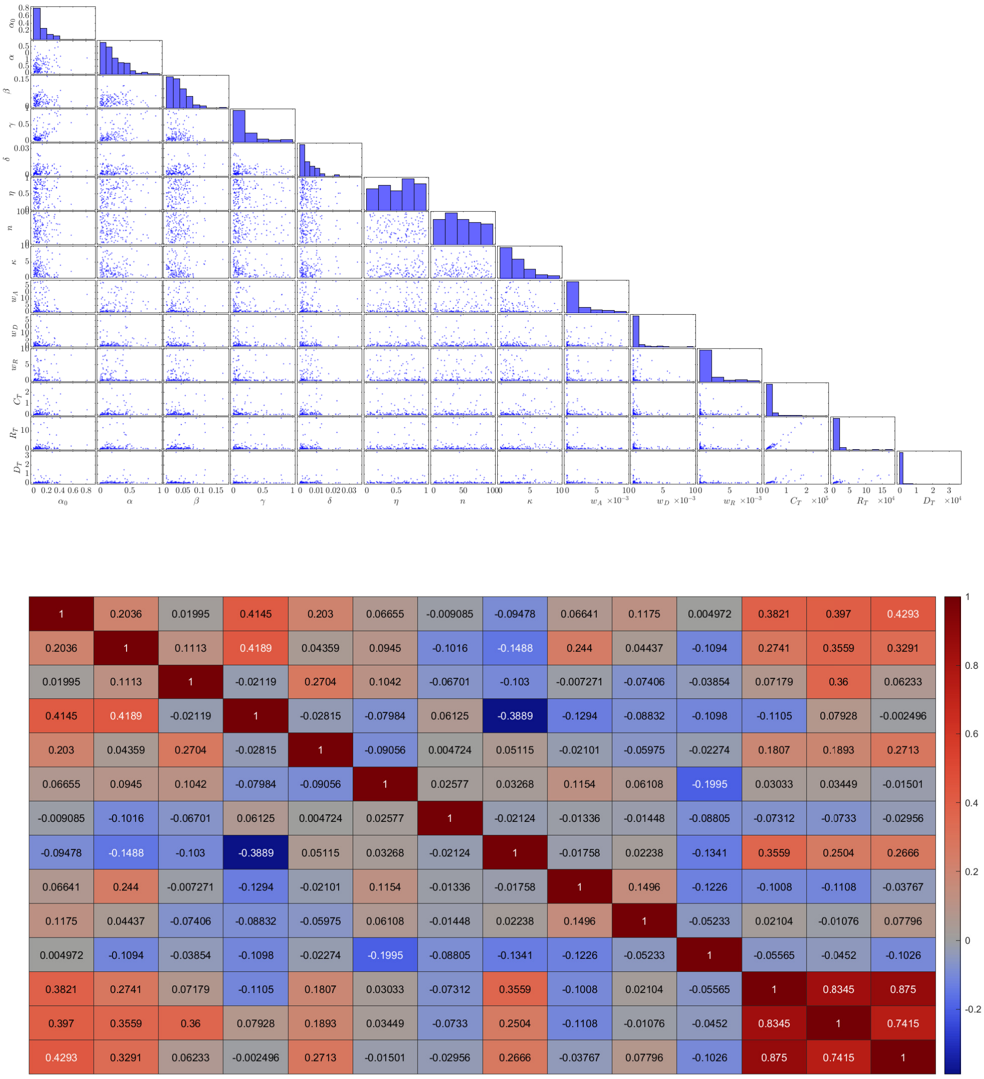

Figure 21: (top) Distributions of model parameter point using full model estimates along with observered cumulative confirmed cases C_T, recoveries R_T and deaths D_T at $T =$ 9 June. (bottom) Spearman correlation coefficients between each point estimate and observed case numbers with the sign and strength of the correlation indicated by the colour-map (positive correlations in red and negative correlations in blue).

References

[1] P. Del Moral, A. Doucet, and A. Jasra. Sequential Monte Carlo samplers. *Journal of the Royal Statistical Society: Series B (Statistical Methodology)*, 68:411–436, 2006.

[2] C. C. Drovandi and A. N. Pettitt. Estimation of parameters for macroparasite population evolution using approximate Bayesian computation. *Biometrics*, 67(1):225–233, 2011.

[3] M. A. Gibson and J. Bruck. Efficient exact stochastic simulation of chemical systems with many species and many channels. *The Journal of Physical Chemistry*, 104:1876–1889, 2000.

[4] D. T. Gillespie. Exact stochastic simulation of coupled chemical reactions. *The Journal of Physical Chemistry*, 81:2340–2361, 1977.

[5] D. T. Gillespie. Approximate accelerated stochastic simulation of chemically reacting systems. *The Journal of Chemical Physics*, 115(4):1716–1733, 2001.

[6] T. G. Kurtz. The relationship between stochastic and deterministic models for chemical reactions. *The Journal of Chemical Physics*,57(7):2976–2978, 1972.

[7] S. A. Sisson, Y. Fan, and M. M. Tanaka. Sequential Monte Carlo without likelihoods. *Proceedings of the National Academy of Sciences of the United States of America*, 104:1760–1765, 2007.

[8] S. A. Sisson, Y. Fan, and M. Beaumont. *Handbook of Approximate Bayesian Computation.* Chapman & Hall/CRC, 1st edition, 2018.

[9] M. Sunnåker, A. G. Busetto, E. Numminen, J. Corander, M. Foll, and C. Dessimoz. Approximate Bayesian computation. *PLOS Computational Biology*, 9:e1002803, 2013.

[10] D. J. Warne, R. E. Baker,and M. J. Simpson. Simulation and inference algorithms for stochastic biochemical reaction networks: form basic concepts to state-of-the-art. *Journal of the Royal Society Interface* 16:20180943, 2019.
